# Chronic kidney disease and undiagnosed atrial fibrillation in individuals with diabetes

**DOI:** 10.1186/s12933-020-01128-y

**Published:** 2020-09-30

**Authors:** Nam Ju Heo, Sang Youl Rhee, Jill Waalen, Steven Steinhubl

**Affiliations:** 1 Department of Internal Medicine, Healthcare System Gangnam Center, Seoul National University Hospital, Seoul, Republic of Korea; 2 Department of Endocrinology and Metabolism, Kyung Hee University School of Medicine, Seoul, Republic of Korea; 3 Scripps Research Translational Institute, La Jolla, CA USA

**Keywords:** Chronic kidney disease, Atrial fibrillation, Diabetes, Wearable ECG patch, Screening for AF, Noninvasive mobile cardiac monitors

## Abstract

**Background:**

Diabetes is an independent risk factor for atrial fibrillation (AF), which is associated with increases in mortality and morbidity, as well as a diminished quality of life. Renal involvement in diabetes is common, and since chronic kidney disease (CKD) shares several of the same putative mechanisms as AF, it may contribute to its increased risk in individuals with diabetes. The objective of this study is to identify the relationship between CKD and the rates of newly-diagnosed AF in individuals with diabetes taking part in a screening program using a self-applied wearable electrocardiogram (ECG) patch.

**Materials and methods:**

The study included 608 individuals with a diagnosis of diabetes among 1738 total actively monitored participants in the prospective mHealth Screening to Prevent Strokes (mSToPS) trial. Participants, without a prior diagnosis of AF, wore an ECG patch for 2 weeks, twice, over a 4-months period and followed clinically through claims data for 1 year. Definitions of CKD included ICD-9 or ICD-10 chronic renal failure diagnostic codes, and the Health Profile Database algorithm. Individuals requiring dialysis were excluded from trial enrollment.

**Results:**

Ninety-six (15.8%) of study participants with diabetes also had a diagnosis of CKD. Over 12 months of follow-up, 19 new cases of AF were detected among the 608 participants. AF was newly diagnosed in 7.3% of participants with CKD and 2.3% in those without (*P *< 0.05) over 12 months of follow-up. In a univariate Cox proportional hazard regression analysis, the risk of incident AF was 3 times higher in individuals with CKD relative to those without CKD: hazard ratios (HR) 3.106 (95% CI 1.2–7.9). After adjusting for the effect of age, sex, and hypertension, the risk of incident AF was still significantly higher in those with CKD: HR 2.886 (95% CI 1.1–7.5).

**Conclusion:**

Among individuals with diabetes, CKD significantly increases the risk of incident AF. Identification of AF prior to clinical symptoms through active ECG screening could help to improve the clinical outcomes in individuals with CKD and diabetes.

## Background

Atrial fibrillation (AF) is the most common arrhythmia in clinical practice resulting in major cardiovascular morbidity and mortality [1]. AF increases the risk of stroke, heart failure and recurrent hospital admissions [2]. For 20% of individuals who experience a stroke due to AF, the occurrence of AF was not diagnosed until the time of their stroke [3]. Opportunistic screening for AF is recommended [4, 5], because if AF is recognized, therapeutic anticoagulation can reduce an absolute risk of all strokes and mortality [6].

The prevalence of AF is particularly high among patients with chronic kidney disease (CKD) [7]. In addition, episodes are more likely to be asymptomatic and the presence of asymptomatic supraventricular arrhythmia including AF was associated with significantly higher risk for mortality in dialysis patients [8]. Identification of preclinical AF may allow earlier opportunities for interventions—those that can prevent or decrease AF episodes such as weight loss and decreasing alcohol intake, or, when appropriate, antithrombotic therapy [9, 10]. It can improve the poor cardiovascular outcomes in patients with CKD. There are a few studies assessing preclinical AF in patients with ESRD requiring hemodialysis [8, 11, 12], and in patients with CKD not receiving dialysis [13, 14]. Recently, with the availability of noninvasive mobile cardiac monitors, there is an opportunity to characterize the incidence and burden of preclinical AF in large populations.

This study aimed to identify the relationship between CKD and the rates of incident AF at 1 year among individuals with diabetes who were enrolled in the prospective mHealth Screening to Prevent Strokes (mSToPS) trial. We focused on individuals with diabetes because diabetes is leading cause of CKD and strong risk for AF [15, 16**].** Studies have demonstrated that patients with diabetes have a 40% higher risk for developing AF relative to patients without diabetes and overall risk increases about 3% per year of diabetes mellitus [17].

## Materials and methods

### Study design

This study included 608 individuals with a diagnosis of diabetes among 1738 total actively monitored participants in the prospective mHealth Screening to Prevent Strokes (mSToPS) trial. The observational cohort study from the mSToPS Trial was approved by the Scripps Office for the Protection of Research Subjects. Participants provided written informed consent digitally. The claims data of this cohort were collected and analyzed as routine for the health plan organization. Protected health information for the observational cohort was not disclosed. The trial was an investigator-initiated trial involving a large health insurance plan’s members throughout the United States. Details of the trial design have been previously published [18, 19].

Eligible individuals were invited by email or direct mail and directed to a web-based informational website if interested in learning more about the study. This site contained detailed information about the study and directed those potentially interested in participating through several high-level screening questions (e.g., confirming health plan membership and no recent AF diagnosis or placement of a pacemaker).

Outcomes data from claims were collected by Healthagen Outcomes, and for ECG patch results, by Scripps Research Translational Institute. Analysis of the combined data was carried out by Healthagen and Scripps Translational Science Institute.

### Participant population

The study population was derived from the Aetna Fully Insured Commercial and Medicare Advantage populations. Inclusion criteria for invitation were developed to include as broad a population as possible that might have an increased likelihood of having undiagnosed AF.

Eligibility for the study included age of 75 years or older, or a male older than age 55 years or female older than 65 years with 1 or more comorbidities listed [19]. Individuals were excluded from the study primarily if they had any current or prior diagnosis of AF, atrial flutter, or atrial tachycardia; were already prescribed anticoagulation therapy; or had an implantable pacemaker, defibrillator, or both; were on dialysis.

Among 1738 patients who wore ECG patch and were monitored actively, 608 patients who have diabetes were included (Additional file 1: Figure S1).

### Study procedures

ECG screening was carried out using the iRhythm Zio XT, a Food and Drug Administration–approved, single-use, water resistant, 14-day, ambulatory ECG monitoring skin adhesive patch that monitors and retains in memory the wearer’s continuous ECG for up to 2 weeks. Participants were asked to wear the patch and then to mail it back to the patch developer via a prepaid mail package. All participants were asked to wear 2 different patches for a period of up to 2 weeks for each patch, each 3 months apart. After participants returned the patch, the rhythm data stored in the device were analyzed using a Food and Drug Administration–approved algorithm. The results then underwent technical review for report generation and quality assurance after which the report was uploaded to a secure website for independent review by the study’s principal investigator. All possible ECG diagnoses of AF were adjudicated, blinded to any diagnosis, by the Clinical Events Adjudication Committee. All ECG patch results were returned to participants at the completion of monitoring. If any potentially actionable results were identified, including a finding of AF, any sustained tachyarrhythmia, or prolonged pause, the participant was contacted by telephone per protocol. After discussion of the findings, the report was sent to the participants and, if they agreed, also was sent to their physician. Participants followed clinically through claims data for 1 year.

### Definitions

AF is defined by ≥ 30 s of AF or flutter detected by device or a new clinical diagnosis recorded in claims data. A diagnosis by claims data required a single entry of an International Classification of Diseases, Ninth Revision (ICD-9) code of 427.3, 427.31, or 427.32, or an International Statistical Classification of Diseases and Related Health Problems, Tenth Revision (ICD-10) code of I48.0, I 48.1, I48.2, I48.3, I48.4, I48.91, or I48.92. Claims-based definitions of a diagnosis of AF included 2 separate ICD-9 or IC10 AF diagnostic codes, and the Health Profile Database algorithm [20, 21]

Definitions of CKD included ICD-9 or ICD-10 chronic renal failure diagnostic codes and the Health Profile Database algorithm using a combination of data types including a serum creatinine [20, 21].

Obesity is defined through the Health Profile Database using a combination of data types including a documented body mass index of 30 or greater and/or an obesity-related diagnosis or procedure (e.g., bariatric surgery).

CHA_2_DS_2_-VAS_C_ score is a clinical prediction score for estimating the risk of stroke in individuals with non-rheumatic atrial fibrillation. An individual’s score can range from 0 to 9, with a high score associated with higher risk. Components include congestive heart failure (1 point); hypertension (1 point); age over 75 years (2 points); diabetes (1 point); prior stroke or transient ischemic attack (2 points); vascular disease (1 point); age 65–74 years (1 point); and sex category (female; 1 point).

### Statistical analysis

In the descriptive analysis of the demographic and clinical characteristics, continuous variables were expressed as the mean and standard deviation, and categorical variables were described numerically with a percentage. Baseline characteristics of CKD group and non-CKD group were compared using *t* tests for continuous variables and χ^2^ or Fisher exact test for categorical variables. Univariate and multivariate logistic regression analysis were performed to assess the effect of CKD on the risk of AF. In multivariate analysis, variables (age, sex, and hypertension) were selected by the stepwise backward methods including variables of P value below 0.05 and excluding variables of P value above 0.10. The data was expressed as hazard ratios (HRs) and 95% confidence intervals. All statistical tests were 2-sided with a significance threshold of *P* < 0.05. The statistical software used was SAS Enterprise Guide version 6.1 (SAS Institute Inc).

## Results

### Overview of cardiac monitoring

A total of 197 individuals wore only one patch, and 411 wore both ECG patches, providing a mean (SD) total wear time of 21.8 days (9.1) per monitored participant with 98.1% analyzable ECG data.

### Characteristics of study participants

The mean (SD) age of study participants was 70.9 (6.7) years and 68.1% were men (Table 1). 5.8% of study participants had a diagnosis of congestive heart failure, 12% of stroke, 5.9% of MI, and 23.8% of patients were diagnosed as obese. The majority of participants were taking antihypertensive medications (93.6%), with 18.9% on beta-blockers. The mean (SD) CHA_2_DS_2_-VAS_C_ score was 3.19 (1.43) years (Table 1).

**Table 1:** Baseline characteristics

Characteristic	No. (%) N = 608
Age, mean (SD), years	70.9 (6.7)
CHA_2_DS_2_-VAS_C_ score, mean (SD)^a^	3.19 (1.43)
Men	414 (68.1)
Atrial fibrillation	19 (3.1)
Atrial fibrillation by claim	14 (2.3)
Wear time per ECG patch, mean (SD), days	11.17 (2.25)
Beta blocker	115 (18.9)
Digoxin	3 (0.5)
Comorbidities	
Hypertension	569 (93.6)
Obesity^b^	145 (23.8)
Heart failure	35 (5.8)
Chronic obstructive pulmonary disease	40 (6.6)
Stroke	73 (12)
Prior myocardial infarction	36 (5.9)
Sleep apnea	171 (28.1)

Results are expressed as frequencies (percentage) and mean values (standard deviation) as appropriate

CHA_2_DS_2_-VAS_C_, congestive heart failure, hypertension, age ≥ 75 years (doubled), diabetes, stroke/transient ischemic attack/thromboembolism (doubled), vascular disease (prior myocardial infarction, peripheral artery disease, or aortic plaque), age 65–75 years, sex category (female)

^a^CHA_2_DS_2_-VAS_C_ score is a clinical prediction score for estimating the risk of stroke in individuals with nonrheumatic atrial fibrillation. An individual’s score can range from 0 to 9, with a high score associated with higher risk. Components include congestive heart failure (1 point); hypertension (1 point); age 75 years (2 points); diabetes (1 point); prior stroke or transient ischemic attack (2 points); vascular disease (1 point); age 65–74 years (1 point); and sex category (female;1 point)

^b^Obesity is defined through the Health Profile Database using a combination of data types including a documented body mass index of 30 or greater and/or an obesity-related diagnosis or procedure (e.g., bariatric surgery)

Baseline characteristics of those with CKD (n = 96, 15.8%) and those without (n = 512, 84.2%) are compared in Table 2. Those with CKD were older and had higher CHA_2_DS_2_-VAS_C_ score. In addition, they had a higher percentage of hypertension, congestive heart failure and chronic obstructive pulmonary disease.

**Table 2:** Baseline characteristics of the participants with and without chronic kidney disease

Characteristic	No (%) Total participants with diabetes (N = 608)		*P*
	Non-chronic kidney disease group	Chronic kidney disease group	
	(N = 512)	(N = 96)	
Age, mean (SD), y	70.4 (6.8)	73.2 (6.0)	< 0.001
CHA_2_DS_2_-VAS_C_ score, mean (SD)^a^	3.09 (1.43)	3.69 (1.34)	< 0.001
Men	356 (69.5)	58 (60.4)	0.052
Atrial fibrillation	12 (2.3)	7 (7.3)	0.02
Atrial fibrillation by claim	8 (1.6)	6 (6.3)	0.014
Beta blocker	101 (19.7)	14 (14.6)	0.149
Digoxin	3 (0.6)	0 (0)	0.597
Comorbidities			
Hypertension	475 (92.8)	94 (97.9)	0.038
Obesity^b^	125 (24.4)	20 (20.8)	0.269
Heart failure	23 (4.5)	12 (12.5)	0.004
Chronic obstructive pulmonary disease	29 (5.7)	11 (11.5)	0.036
Stroke	65 (12.7)	8 (8.3)	0.149
Prior myocardial infarction	27 (5.3)	9 (9.4)	0.097
Sleep apnea	143 (27.9)	28 (29.2)	0.446

Results are expressed as frequencies (percentage) and mean values (standard deviation) as appropriate

CHA_2_DS_2_-VAS_C_, congestive heart failure, hypertension, age ≥ 75 years (doubled), diabetes, stroke/transient ischemic attack/thromboembolism (doubled), vascular disease (prior myocardial infarction, peripheral artery disease, or aortic plaque), age 65–75 years, sex category (female)

^a^CHA_2_DS_2_-VAS_C_ score is a clinical prediction score for estimating the risk of stroke in individuals with nonrheumatic atrial fibrillation. An individual’s score can range from 0 to 9, with a high score associated with higher risk. Components include congestive heart failure (1 point); hypertension (1 point); age 75 years (2 points); diabetes (1 point); prior stroke or transient ischemic attack (2 points); vascular disease (1 point); age 65–74 years (1 point); and sex category (female;1 point)

^b^Obesity is defined through the Health Profile Database using a combination of a data types including a documented body mass index of 30 or greater and/or an obesity-related diagnosis or procedure (e.g., bariatric surgery)

### Characteristics of atrial fibrillation

Over 12 months of follow-up, 19 new cases of AF were detected among 608 patients (3.1 per 100 person-years). Only 2 experienced symptoms which were mild and did not lead to a clinical evaluation. Among the newly diagnosed cases, 5 individuals (26%) were first found to have AF by ECG patch and 14 individuals received a clinical diagnosis of AF either prior to monitoring or after monitoring was completed without any findings of AF during monitoring.

The median duration of an individual’s longest duration of AF was 309 min (IQR, 17.2–707 min). The median burden of AF (percentage of monitored time in AF) was 2.5% (IQR,<1–6%).

### CKD and atrial fibrillation

**Fig. 1:**
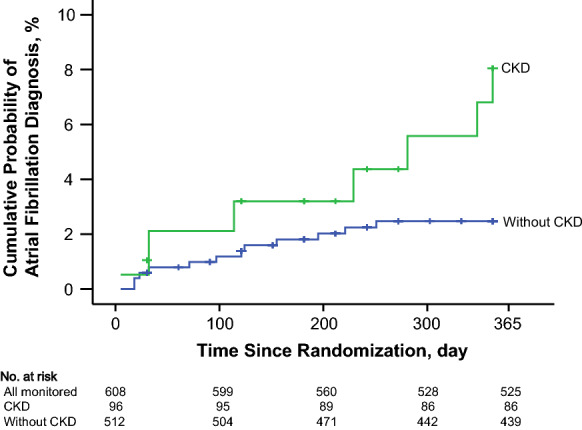
Cumulative rate of first diagnosis of atrial fibrillation in the participants with and without chronic kidney disease

Over 12 months of follow-up, 7 cases of AF were newly diagnosed among 96 participants with CKD (7.3 per 100 person-years). Among 512 participants without CKD, 12 new cases of AF were detected (2.3 per 100 person-years) (*P *< 0.05, Table 2 and Fig. 1).

In a univariate Cox proportional hazard regression analysis, the risk of incident AF was 3 times higher in the patients with CKD than in those without: hazard ratio (95% confidence intervals) was 3.106 (1.223 to 7.890) (Table 3). After adjusting for the effect of age and sex (Model 1) or hypertension (Model 2), the risk of incident AF was still significantly higher in participants with diabetes and CKD relative to those with diabetes and no CKD: hazard ratios (95% confidence intervals) was 2.886 (1.106 to 7.529) (Table 3).

**Table 3:** Cox proportional hazard regression analysis of CKD for the risk of AF

	Univariate		Multivariate			
	HR (95% CI)	*P*	Model 1^a^		Model 2^b^	
			HR (95% CI)	*P*	HR (95% CI)	*P*
CKD	3.106 (1.223–7.89)	0.017	2.903 (1.122–7.511)	0.028	2.886 (1.106–7.529)	0.030

*CKD* chronic kidney disease, *CI* confidence internal, *HR* hazard ratio, *AF* atrial fibrillation

^a^Model 1 was adjusted for age and sex

^b^Model 2 was adjusted for age, sex and hypertension

## Discussion

In this study, we investigated the relationship between AF and CKD in individuals with diabetes using wearable ECG patch. Our results demonstrate that CKD increases the risk for AF significantly, even in a non-dialysis-requiring CKD population.

To the best of our knowledge, this study is the first prospective large-scale investigation of screening an asymptomatic cohort for AF reporting that having CKD significantly increases the risk of AF among individuals with diabetes. We used noninvasive, wearable and clinically available patch to detect even preclinical cardiac AF.

There are a few studies assessing preclinical AF using implantable cardiac monitors or loop recorders in patients with ESRD requiring hemodialysis [8, 11]. In a recent paper of 66 patients on dialysis, 41% had AF with temporal associations with timing of dialysis [12]. A study of 77 dialysis patients also found higher rates of supraventricular arrhythmias including AF during dialysis [8]. Although these studies are informative, because they have not provided data on the larger, non-dialysis-requiring CKD population, these findings are difficult to apply to general CKD population. Although there is only one study reporting preclinical AF in the 38 patients with CKD not requiring dialysis and diabetes using mobile cardiac telemetry monitors [14], there is no data on the large, non-dialysis-requiring CKD population until now. In the current study, we compared the incidence rate of AF using wearable ECG patch among the 96 participants with CKD who are not requiring dialysis versus 512 participants without CKD, unlike previous studies without normal control.

Previous studies have reported that the incidence of AF was increased in individuals with end-stage renal disease and glomerular filtration rate inversely and significantly associated with the rate of incident AF [12, 13, 22, 23]. Recent study reported that the incidence rates of AF were 12.1 and 7.3 per 1000 person-years for end-stage renal disease and CKD patients, respectively. The prevalence was lower than that of our study because we detected even preclinical cardiac AF using wearable ECG patch. In our study, CKD was significantly associated with greater risk of incident AF among individuals with diabetes, which was consistent with previous reports. Akoum et al. [14] reported that 11% of newly diagnosed AF for 11.2 days of monitoring period using ambulatory cardiac monitoring among 38 patients with CKD and diabetes. It is higher than the result of our study that 7.3% of newly diagnosed AF was reported for 1 year of follow up period. The difference might be caused by the difference of the population. In that study, 18% of participants had a history of AF and their mean duration of diabetes was relatively long as 20.1 years. In our study, however, participants with history of AF was excluded.

In our study, 17 patients among 19 new cases of AF did not have any symptom and 2 patients experienced only mild symptoms that didn’t cause them to seek medical care. Similarly, in other study reporting the newly diagnosed AF among patients with CKD and diabetes using ambulatory cardiac monitoring none of the newly diagnosed patients have experienced any symptoms [14]. In study of 77 dialysis patients, 38% of supraventricular arrhythmias (SVA) including 14% of AF were observed during dialysis, which were all asymptomatic and the SVA associated with a fourfold increased risk of cardiovascular events. Patients with SVA also had a higher risk of nonfatal cardiovascular events (hazard ratio, 4.32; 95% CI 2.1 to 8.8) and symptomatic AF during follow-up (hazard ratio, 17.19; 95% CI 2.03 to 145.15) [8]. Asymptomatic AF is common. Boriani et al. [24] reported that asymptomatic AF accounted for 39.7% of all AF and mortality at 1 year was more than twofold higher in asymptomatic patients compared with symptomatic patients (9.4% vs 4.2%, *P* < 0.001) and was associated independently with CKD. Although the prevalence of diabetes was higher in asymptomatic AF patients compared with symptomatic patients, it was not statistically significant. Identification of subclinical AF with wearable ECG patch or other active screening program may improve cardiovascular outcomes in patients with diabetes and CKD, because subclinical AF could be important precursors to significant cardiovascular events.

Five patients among 19 new cases of AF were diagnosed by ECG patch and 14 patients were diagnosed by claim in this study. Among participants with CKD, only one patient (14%) was diagnosed by ECG patch and the other 6 patients (86%) were diagnosed clinically and identified in claims data. It is possible that the reason that the frequency of clinical diagnosis rather than a diagnosis by ECG patch was higher in individuals with CKD relative to those without is that patients with CKD likely have more frequent health system contact than those without. They were older (age 73.2 vs 70.4), had a higher CHA_2_DS_2_-VAS_C_ score (3.69 vs 3.09), more congestive heart failure (12.5% vs 4.5%) and chronic obstructive pulmonary disease (11.5% vs 5.7%).

Renal dysfunction has been documented to be associated with AF [25]. Several studies have reported risk factors associated with new-onset AF in patients with CKD included age and comorbid diseases such as diabetes mellitus, hypertension, heart failure and coronary artery disease [26, 27]. These are well-known risk factors of kidney disease and also considered to be related to electrical and structural remodeling of the atria, which could have an important role in the promotion of AF [28, 29]. These risk factors could be associated with insulin resistance (IR). IR is a condition in which cells fail to respond normally to insulin, and characterized by a set of signs comprising obesity, increased blood sugar, dyslipidemia, and elevated blood pressure. IR could be considered as a risk factor for kidney disease and AF and plausible mechanism of both kidney disease and AF [30–32].

Regarding the value of active screening for AF, further work is needed to help better understand the implications of preclinical AF, its risks and the value of various therapeutic interventions. Identification of preclinical AF may allow earlier opportunities for interventions. There are evidence based therapies that decrease or eliminate future AF such as weight loss [8], alcohol abstinence [9], and sleep apnea treatment [33], as well as unknown but possible value of initiating anticoagulation therapy. It can improve the poor cardiovascular outcomes in patients with CKD. The clinical value of active screening for AF will be informed through follow up on studies such as STROKESTOP and mSToPS trial.

This study has several limitations. First, we could not analyze the exact correlation between the renal function and the risk of AF, because we could not get serum creatinine data from Aetna the insurance company. Instead, ICD codes and the Health Profile Database algorithm were used to define CKD. Second, only participants with diabetes was included in this study. Although diabetes is correlated with AF and CKD closely, the findings may not be generalizable to general population. Third, clinical outcome data were not included in this analysis but will be reported in a planned 3-year follow-up of mSToPS trial participants.

## Conclusions

In conclusion, in this study of participants with diabetes, we found CKD could be associated with an increased risk of incident AF. Because most of these individuals experienced no or little symptoms, identification of subclinical episodes of AF with a wearable ECG patch or other active screening program might be especially valuable in this population. Further data are needed to determine how the identification of subclinical AF can drive therapeutic interventions that reduce cardiovascular complications and improve overall survival.

## **Supplementary information**

**Additional file 1: Figure S1.** Participant flow diagram.

## Data Availability

The datasets used and/or analyzed during the current study are available from the corresponding author on reasonable request.

## Abbreviations

AF: Atrial fibrillation
ECG: Electrocardiogram
CKD: Chronic kidney disease
ICD-9: International Classification of Diseases, Ninth Revision
ICD-10: International Statistical Classification of Diseases and Related Health Problems, Tenth Revision

## References

[1] Ball J, Carrington MJ, McMurray JJ, Stewart S (2013). Atrial fibrillation: profile and burden of an evolving epidemic in the 21st century. Int J Cardiol.

[2] Benjamin EJ, Levy D, Vaziri SM, D’Agostino RB, Belanger AJ, Wolf PA (1994). Independent risk factors for atrial fibrillation in a population-based cohort. The Framingham Heart Study. JAMA.

[3] Lin HJ, Wolf PA, Benjamin EJ, Belanger AJ, D’Agostino RB (1995). Newly diagnosed atrial fibrillation and acute stroke: the Framingham Study. Stroke.

[4] Goldstein LB, Bushnell CD, Adams RJ, Appel LJ, Braun LT, Chaturvedi S, Creager MA, Culebras A, Eckel RH, Hart RG (2011). American Heart Association Stroke Council; Council on Cardiovascular Nursing; Council on Epidemiology and Prevention; Council for High Blood Pressure Research; Council on Peripheral Vascular Disease, and Interdisciplinary Council on Quality of Care and Outcomes Research. Guidelines for the primary prevention of stroke. Stroke..

[5] Kirchhof P, Benussi S, Kotecha D, Ahlsson A, Atar D, Casadei B, Castella M, Diener HC, Heidbuchel H, Hendriks J (2016). ESC Scientific Document Group. 2016 ESC Guidelines for the management of atrial fibrillation developed in collaboration with EACTS. Eur Heart J..

[6] Hart RG, Pearce LA, Aguilar MI (2007). Meta-analysis: antithrombotic therapy to prevent stroke in patients who have nonvalvular atrial fibrillation. Ann Intern Med.

[7] Collins AJ, Foley RN, Herzog C, Chavers B, Gilbertson D, Herzog C, Ishani A, Johansen K, Kasiske B, Kutner N, et al. US renal data system 2012 annual data report. Am J Kidney Dis. 2013; 61(1 Suppl 1): A7, e1-476. 10.1053/j.ajkd.2012.11.031 23253259

[8] Verde E, Pérez de Prado A, López-Gómez JM, Quiroga B, Goicoechea M, García-Prieto A, Torres E, Reque J, Luño J (2016). Asymptomatic intradialytic supraventricular arrhythmias and adverse outcomes in patients on hemodialysis. Clin J Am Soc Nephrol..

[9] Middeldorp ME, Pathak RK, Meredith M, Mehta AB, Elliott AD, Mahajan R, Twomey D, Gallagher C, Hendriks JML, Linz D (2018). PREVEntion and regReSsive Effect of weight-loss and risk factor modification on Atrial Fibrillation: the REVERSE-AF study. Europace..

[10] Voskoboinik A, Kalman JM, De Silva A, Nicholls T, Costello B, Nanayakkara S, Prabhu S, Stub D, Azzopardi S, Vizi D (2020). Alcohol abstinence in drinkers with atrial fibrillation. N Engl J Med.

[11] Roberts PR, Zachariah D, Morgan JM, Yue AM, Greenwood EF, Phillips PC, Kalra PA, Green D, Lewis RJ, Kalra PR (2017). Monitoring of arrhythmia and sudden death in a hemodialysis population: the CRASH-ILR Study. PLoS ONE.

[12] Roy-Chaudhury P, Tumlin JA, Koplan BA, Costea AI, Kher V, Williamson D, Pokhariyal S, Charytan DM, Di M (2018). MiD Investigators and Committees: primary outcomes of the Monitoring in Dialysis Study indicate that clinically significant arrhythmias are common in hemodialysis patients and related to dialytic cycle. Kidney Int.

[13] Bansal N, Zelnick LR, Alonso A, Benjamin EJ, de Boer IH, Deo R, Katz R, Kestenbaum B, Mathew J, Robinson-Cohen C (2017). eGFR and albuminuria in relation to risk of incident atrial fibrillation: a meta-analysis of the Jackson Heart Study, the Multi-Ethnic Study of Atherosclerosis, and the Cardiovascular Health Study. Clin J Am Soc Nephrol.

[14] Akoum N, Zelnick LR, de Boer IH, Hirsch IB, Trence D, Henry C, Robinson N, Bansal N (2019). Rates of cardiac rhythm abnormalities in patients with CKD and diabetes. Clin J Am Soc Nephrol.

[15] Tadic M, Cuspidi C (2015). Type 2 diabetes mellitus and atrial fibrillation: from mechanisms to clinical practice. Arch Cardiovasc Dis..

[16] Kim YG, Han K, Choi J, Boo KY, Kim DY, Oh SK, Lee KN, Shim J, Kim JS, Kim Y (2019). The impact of body weight and diabetes on new-onset atrial fibrillation: a nationwide population based study. Cardiovasc Diabetol..

[17] Dublin S, Glazer NL, Smith NL, Psaty BM, Lumley T, Wiggins KL, Page RL, Heckbert SR (2010). Diabetes mellitus, glycemic control, and risk of atrial fibrillation. J Gen Intern Med.

[18] Steinhubl SR, Mehta RR, Ebner GS, Ballesteros MM, Waalen J, Steinberg G, Van Crocker P, Felicione E, Carter CT, Edmonds S (2016). Rationale and design of a home-based trial using wearable sensors to detect asymptomatic atrial fibrillation in a targeted population: the mHealth Screening to Prevent Strokes (mSToPS) trial. Am Heart J.

[19] Steinhubl SR, Waalen J, Edwards AM, Ariniello LM, Mehta RR, Ebner GS, Carter C, Baca-Motes K, Felicione E, Sarich T (2018). Effect of a home-based wearable continuous ecg monitoring patch on detection of undiagnosed atrial fibrillation: the mSToPS Randomized Clinical Trial. JAMA.

[20] Hanchak NA, Murray JF, Hirsch A, McDermott PD, Schlackman N (1996). USQA health profile database as a tool for health plan quality improvement. Manag Care..

[21] Jensen PN, Johnson K, Floyd J, Heckbert SR, Carnahan R, Dublin S (2012). A systematic review of validated methods for identifying atrial fibrillation using administrative data. Pharmacoepidemiol Drug Saf.

[22] Wizemann V, Tong L, Satayathum S, Disney A, Akiba T, Fissell RB, Kerr PG, Young EW, Robinson BM (2010). Atrial fibrillation in hemodialysis patients. Clinical features and associations with anticoagulant therapy. Kidney Int..

[23] Wetmore JB, Mahnken JD, Rigler SK, Ellerbeck EF, Mukhopadhyay P, Spertus JA, Hou Q, Shireman TI (2012). The prevalence of and factors associated with chronic atrial fibrillation in medicare/Medicaid eligible dialysis patients. Kidney Int.

[24] Boriani G, Laroche C, Diemberger I, Fantecchi E, Popescu MI, Rasmussen LH, Sinagra G, Petrescu L, Tavazzi L, Maggioni AP, Lip GY (2015). Asymptomatic atrial fibrillation: clinical correlates, management, and outcomes in the EORP-AF Pilot General Registry. Am J Med.

[25] Ahmadi SS, Svensson A, Pivodic A, Rosengren A, Lind M (2020). Risk of atrial fibrillation in persons with type 2 diabetes and the excess risk in relation to glycemic control and renal function: a Swedish cohort study. Cardiovasc Diabetol..

[26] Liao J, Chao T, Liu C, Wang K, Chen S, Lin Y, Chang S, Lo L, Hu Y, Tuan T, Chung F, Chen T, Chen S (2015). Incidence and risk factors for new-onset atrial fibrillation among patients with end-stage renal disease undergoing renal replacement therapy. Kidney Int.

[27] Bansal N, Fan D, Hsu C, Ordonez J, Marcus G, Go AS (2013). Incident atrial fibrillation and risk of end-stage renal disease in adults with chronic kidney disease. Circulation.

[28] Tuan TC, Chang SL, Tsao HM (2010). The impact of age on the electroanatomical characteristics and outcome of catheter ablation inpatients with atrial fibrillation. J Cardiovasc Electrophysiol.

[29] Hsieh MH, Lin YJ, Wang HH (2013). Functional characterization of atrial electrograms in a pacing-induced heart failure model of atrial fibrillation: importance of regional atrial connexin40 remodeling. J Cardiovasc Electrophysiol.

[30] Chan YH, Chang GJ, Lai YJ, Chen WJ, Chang SH, Hung LM, Kuo CT, Yeh YH (2019). Atrial fibrillation and its arrhythmogenesis associated with insulin resistance. Cardiovasc Diabetol..

[31] Fontes JD, Lyass A, Massaro JM, Rienstra M, Dallmeier D, Schnabel RB, Wang TJ, Vasan RS, Lubitz SA, Magnani JW (2012). Insulin resistance and atrial fibrillation (from the Framingham Heart Study). Am J Cardiol.

[32] Cho ME, Craven TE, Cheung AK, Glasser SP, Rahman M, Soliman EZ, Stafford RS, Johnson KC, Bates JT, Burgner A (2017). The association between insulin resistance and atrial fibrillation: a cross-sectional analysis from SPRINT (Systolic Blood Pressure Intervention Trial). J Clin Hypertens (Greenwich)..

[33] Gami AS, Hodge DO, Herges RM, Olson EJ, Nykodym J, Kara T, Somers VK (2007). Obstructive sleep apnea, obesity, and the risk of incident atrial fibrillation. J Am Coll Cardiol..

