# Supplementary figures and images for: Chronic kidney disease and undiagnosed atrial fibrillation in individuals with diabetes
Source: Cardiovasc Diabetol. 2020 Sep 30;19:157. doi: 10.1186/s12933-020-01128-y (PMC7528591; doi:10.1186/s12933-020-01128-y)

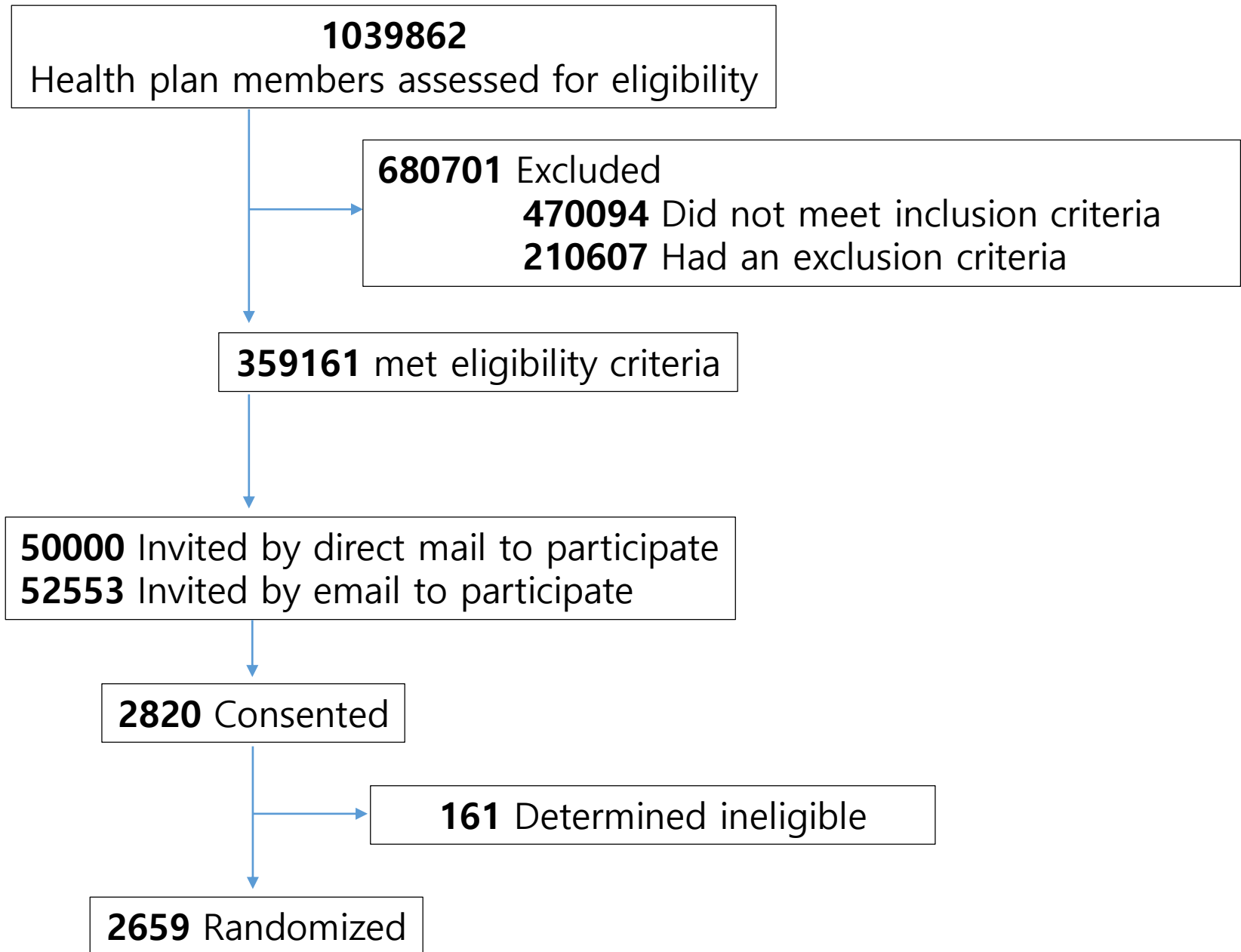

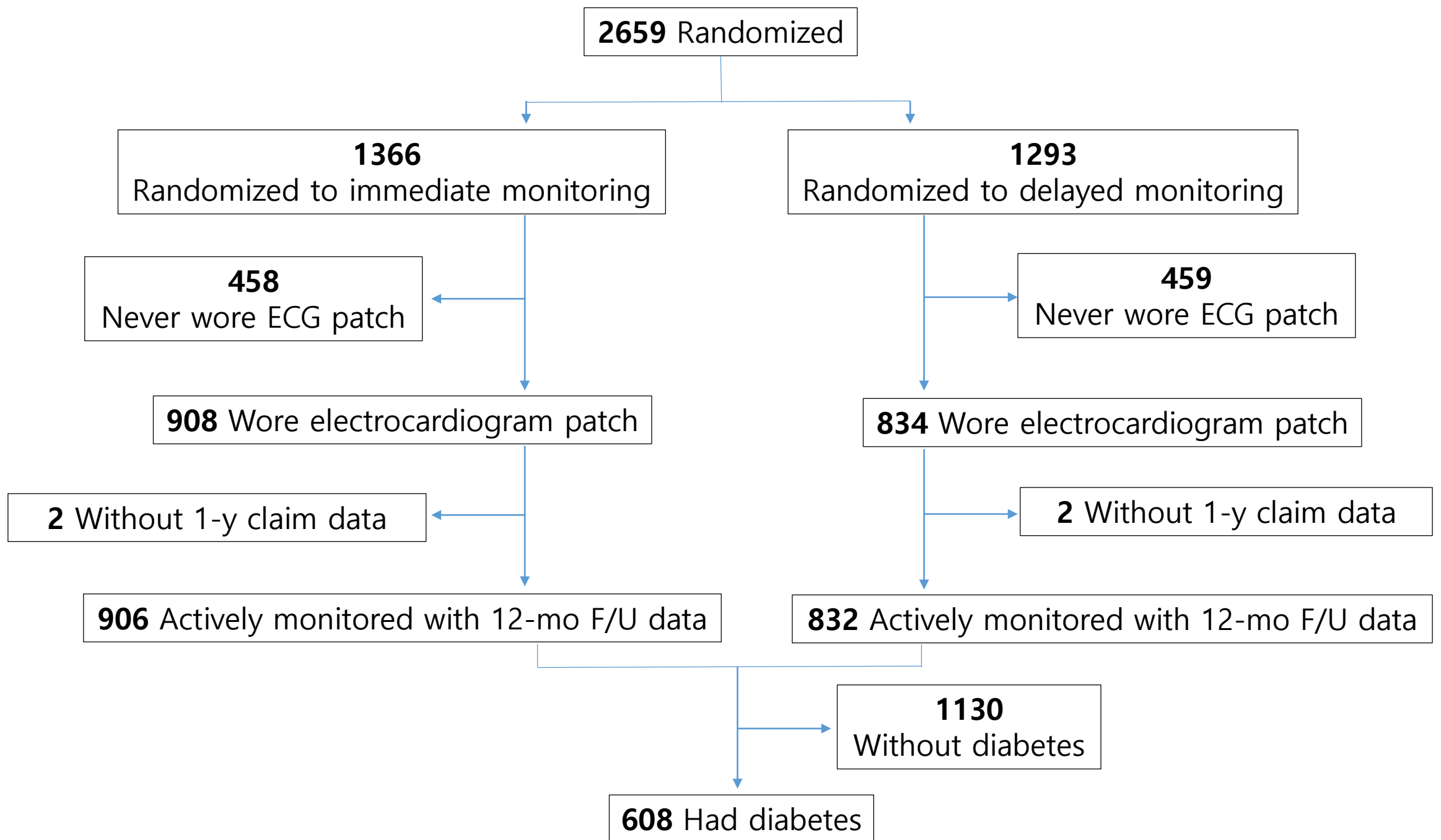

Supplement: Supplementary file 1 — Additional file 1: Figure S1. Participant flow diagram. [file 12933_2020_1128_MOESM1_ESM.pdf]
